# Proprioceptive short-term memory in passive motor learning

**DOI:** 10.1038/s41598-023-48101-9

**Published:** 2023-11-27

**Authors:** Shinya Chiyohara, Jun-ichiro Furukawa, Tomoyuki Noda, Jun Morimoto, Hiroshi Imamizu

**Affiliations:** 1 Brain Information Communication Research Laboratory Group, Advanced Telecommunications Research Institute International (ATR), Keihanna Science City, Kyoto, 619-0288 Japan; 2 Man-Machine Collaboration Research Team, Guardian Robot Project, RIKEN, Kyoto, Japan; 3 Graduate School of Informatics, Kyoto University, Kyoto, Japan; 4 Department of Psychology, Graduate School of Humanities and Sociology, The University of Tokyo, Hongo 7-3-1, Bunkyo-Ku, Tokyo, 113-0033 Japan; 5 Research Into Artifacts, Center for Engineering, School of Engineering, The University of Tokyo, Hongo 7-3-1, Bunkyo-Ku, Tokyo, 113-8656 Japan

**Keywords:** Cognitive neuroscience, Perception, Human behaviour, Motor control, Short-term memory

## Abstract

A physical trainer often physically guides a learner’s limbs to teach an ideal movement, giving the learner proprioceptive information about the movement to be reproduced later. This instruction requires the learner to perceive kinesthetic information and store the instructed information temporarily. Therefore, (1) proprioceptive acuity to accurately perceive the taught kinesthetics and (2) short-term memory to store the perceived information are two critical functions for reproducing the taught movement. While the importance of proprioceptive acuity and short-term memory has been suggested for active motor learning, little is known about passive motor learning. Twenty-one healthy adults (mean age 25.6 years, range 19–38 years) participated in this study to investigate whether individual learning efficiency in passively guided learning is related to these two functions. Consequently, learning efficiency was significantly associated with short-term memory capacity. In particular, individuals who could recall older sensory stimuli showed better learning efficiency. However, no significant relationship was observed between learning efficiency and proprioceptive acuity. A causal graph model found a direct influence of memory on learning and an indirect effect of proprioceptive acuity on learning via memory. Our findings suggest the importance of a learner’s short-term memory for effective passive motor learning.

## Introduction

**Figure 1:**
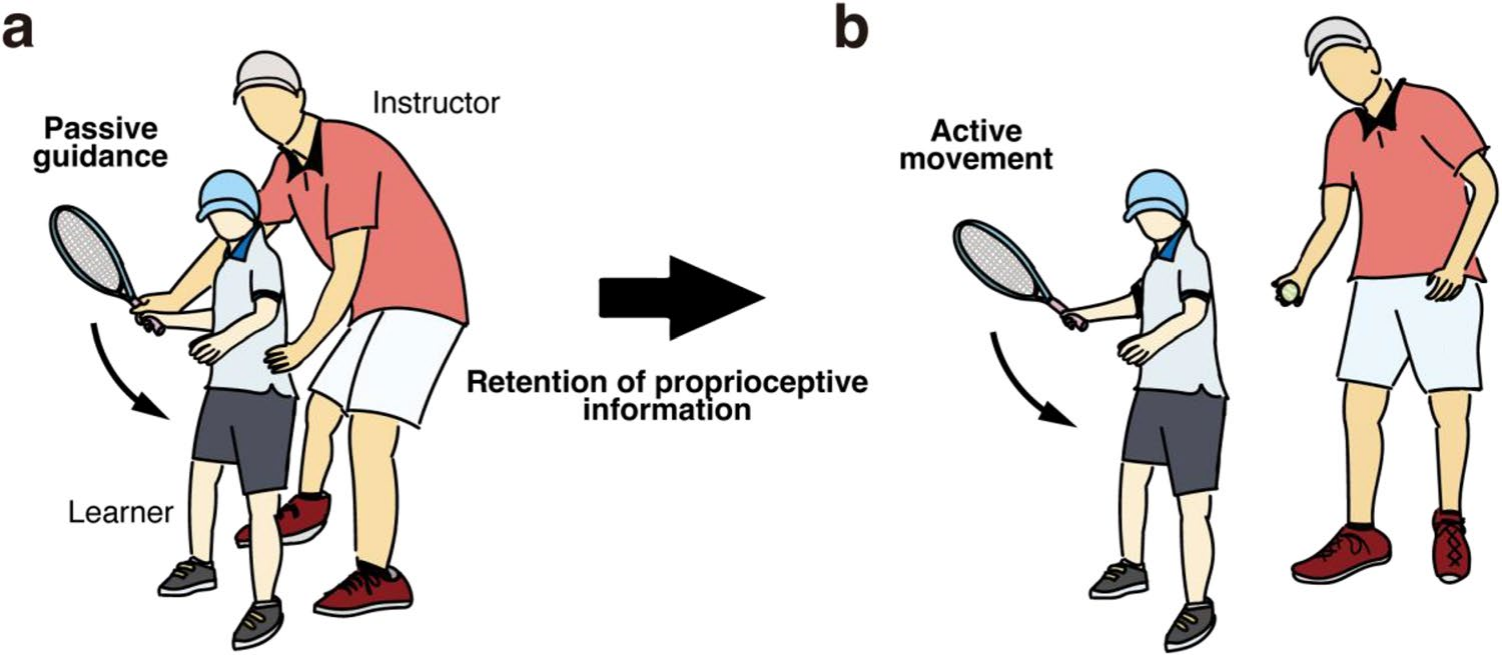
Example of passive physical guidance of movement. (**a**) A sports instructor grasps the learner’s body and guides movements to teach a swing form through proprioception. (**b**) The learner then actively swings the racket while recalling the sensory experiences provided by the instructor.

When teaching a new motor skill, such as a racket swing form (Fig. 1a), a sports instructor may grasp the learner's body and guide movements to teach the ideal movement (a correct form) through proprioception^1,2^. This type of passive physical guidance is common in sports instruction, parent–child interactions, and physical rehabilitation (therapist-client interaction). The physical guidance provides the learner with proprioceptive information about the movement to be subsequently reproduced^3^. Consequently, the learner must accurately acquire proprioceptive information on the instructed movement and then memorize/retain that information until they make the movement (Fig. 1b). Thus, there seems be two critical elements to this type of learning: proprioceptive acuity and short-term memory. Proprioceptive acuity is the ability to detect subtle differences between movements, and this ability affects learning efficiency^4,5^. If a learner has poor proprioceptive acuity, that learner may gain little information from the passive physical guidance (Fig. 1a). Short-term proprioceptive memory is the ability to temporarily store proprioceptive information. If a learner has poor short-term memory capacity, that learner may retain little information.

Regarding proprioception, loss or decline of proprioceptive function leads to problems transferring proprioceptive information to the central nervous system. For instance, patients with deafferentation have difficulty controlling body movements and motor adaptation without visual feedback^6^. Several studies^4,5^ have suggested that aging-related decline in proprioceptive function^7–9^ results in poor motor learning. However, these studies investigated the contribution of proprioception to active motor learning in patients or older adults. As a result, in healthy adults, it is not yet fully understood whether proprioceptive acuity directly influences learning efficiency in passive motor learning through physical guidance.

Regarding short-term memory, previous studies have highlighted the importance of “visuospatial” short-term memory in the early stages of active motor learning^10–14^. By contrast, few studies have investigated the role of “proprioceptive” short-term memory in motor learning. A previous study^15^ used a proprioceptive memory task in which participants remembered the direction of passive arm movements guided by a robotic device. This study found that participants with better proprioceptive memory performance showed more significant motor learning when reaching for a hidden target actively. However, as mentioned above, little is known about the relationship between proprioceptive memory and passive motor learning, in which the retention of proprioceptive information is crucial for reproducing the passively guided movement (Fig. 1b).

In this study, we hypothesize that an individual's proprioceptive acuity and short-term memory capacity are associated with learning efficiency in passively guided training. Specifically, we hypothesize that learners with better proprioceptive acuity and short-term memory can learn new skills more efficiently. To test this hypothesis, we measured participants’ proprioceptive acuity, performance on a short-term memory task (modified from Ref.^15^), and performance in a passively guided motor learning task^3^. In a task measuring proprioceptive acuity, participants were required to detect subtle differences between the elbow joint angles presented sequentially by the robotic device. In the short-term memory task, we assessed participants’ ability to remember three elbow joint angles presented by the device. In the motor learning task, the device moved the participants’ forearms to instruct them in a continuous pattern of elbow joint movement for 10 s, and participants reproduced the taught movement as accurately as possible. We found that the learning efficiency in the passive training was more significant for participants with better proprioceptive short-term memory. By contrast, we did not find a significant correlation between an individual’s acuity and efficiency in the above motor learning. Furthermore, we used a linear non-Gaussian model^16^ to estimate the causal relationship among proprioceptive acuity, short-term memory, and motor learning efficiency. As a result, we found that short-term memory performance, rather than acuity, affected learning efficacy. Our results suggest that the learner’s proprioceptive short-term memory capacity must be considered in designing effective learning by passively guided physical training.

## Results

**Figure 2:**
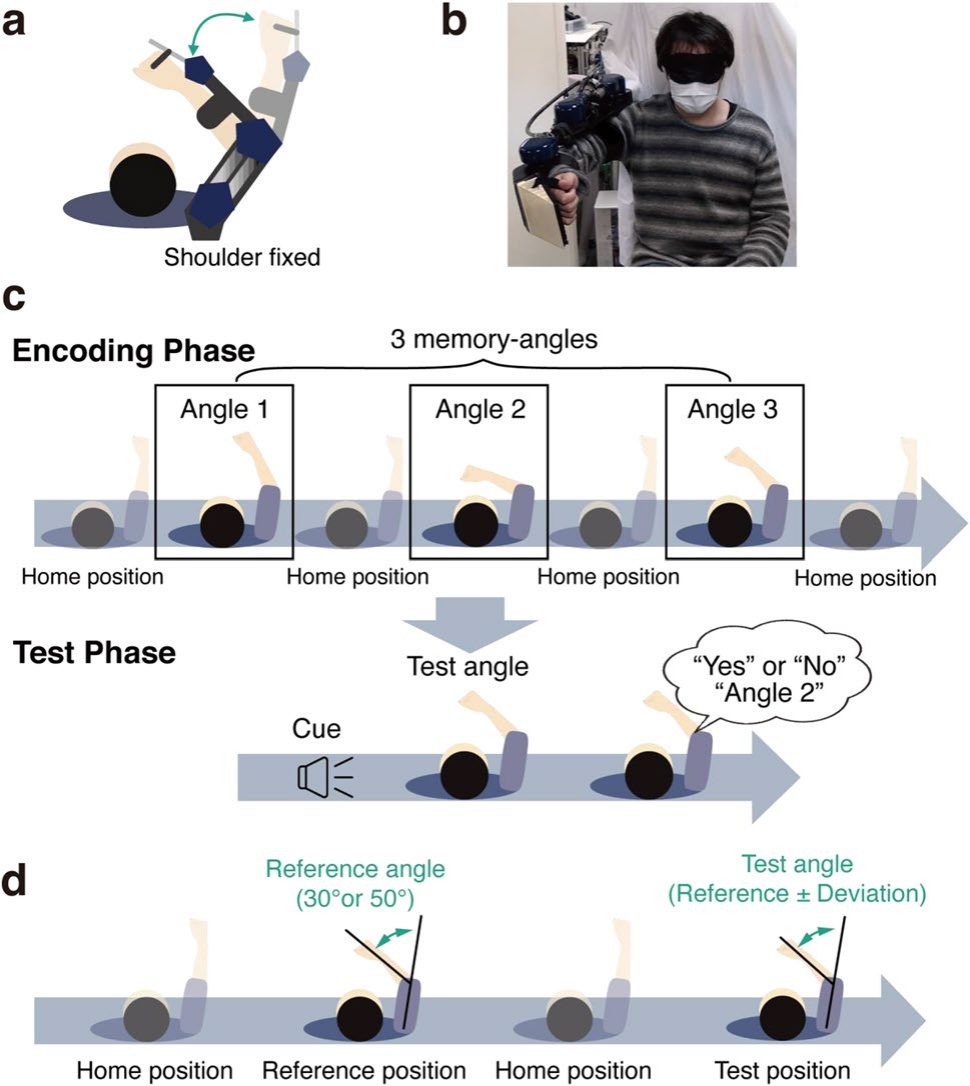
Experimental setup and tasks to measure participants’ proprioceptive performance. (**a**) An exoskeleton robot guided the participant’s elbow joint in flexion and extension directions in the horizontal plane. (**b**) Photographs of the exoskeleton robot from the frontal view. (**c**) Proprioceptive short-term memory task. (**d**) Proprioceptive judgment task.

**Figure 3:**
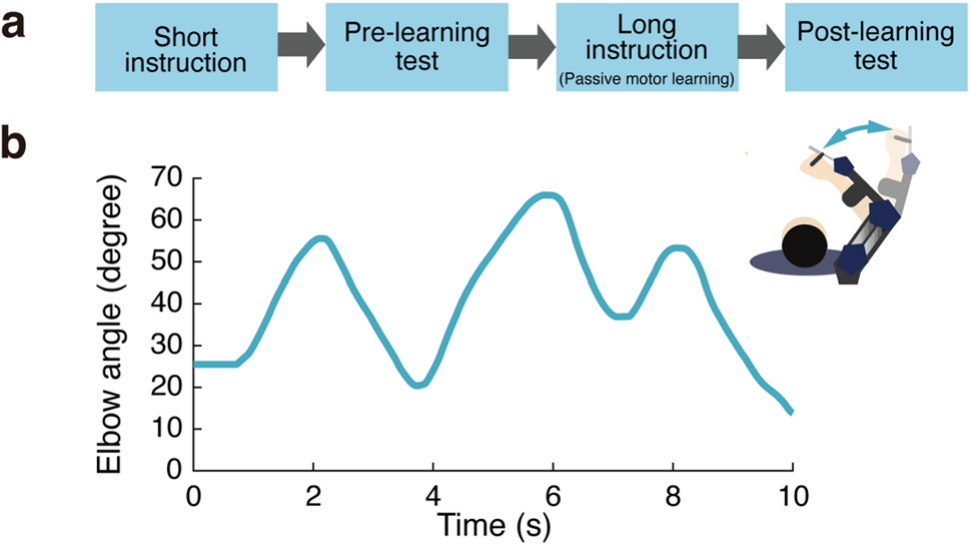
Passive physical guidance task. (**a**) Task procedure (**b**) Target trajectory of elbow joint, along which the exoskeleton robot moved the participant’s forearm (inset) during the instruction periods.

Twenty-one healthy right-handed participants took part in the experiment. We used an upper limb exoskeleton robot^17,18^ to move the forearm and elbow joints (Fig. 2a,b). Participants performed three tasks: (1) a proprioceptive short-term memory task (Fig. 2c), (2) a proprioceptive judgment task (Fig. 2d), and (3) a passive physical guidance task (Fig. 3). To avoid the learning effect of passive physical guidance, the three tasks were performed in the above order by all participants. During the tasks, participants’ eyes were occluded by an eye mask to prevent visual identification of the arm position.

### Proprioceptive short-term memory

A short-term memory task was adopted from a previous study^15^ and modified for the exoskeleton robot. This task consisted of 60 trials. Figure 2c shows the timeline of each trial. At the beginning of each trial, the robot extended the participant's right elbow joint to align the forearm and upper arm in a straight line (the home angle: 0°). The robot then flexed the elbow joint from the home angle to one of the three memory angles in 2 s and held it for 2 s before returning to the home angle. This sequence was repeated three times during the encoding phase. Participants were instructed to maintain a relaxed state and to memorize the three angles. The encoding phase was followed by a test phase in which the robot flexed the elbow joint to a test angle after a beep sound. After the test angle was given, they were asked to verbally respond whether the test angle was "present" or "absent" among the three memorized angles. If the response was “present”, the participant then responded with which remembered angle matched the test angle. We decided the order of “present” and “absent” conditions pseudo-randomly. The three elbow joint angles ranged from 8° to 82°, each presented with a difference from the previous one of more than 10°. In half of the trials (30 trials), the test angle was one of the three memorized angles. In the remaining 30 trials, the test angle was a new angle that differed by more than 10° from one of the three memorized angles (see Methods for details). The order of the test angles was also pseudo-random.

To estimate the short-term memory performance, we first counted hits, misses, false alarms, and correct rejections from the response patterns to the presence/absence of the test angle. Then, we calculated the sensitivity (hit/(hit + miss)) and precision (hit/(hit + false alarm)) based on these data. In addition, the d-prime (Z(hit rate) − Z(false alarm rate)) was calculated using signal detection theory. The overall sensitivity was 0.84 (SD 0.11), while the precision was 0.68 (SD 0.05). The d-prime was 1.39 (SD 0.53).

### Proprioceptive acuity

We performed a proprioceptive judgment task using the exoskeleton robot to estimate the proprioceptive acuity. The task consisted of 80 trials. Figure 2d shows the timeline used in each trial. At the beginning of each trial, the participant's elbow joint was extended to the home angle. The robot moved the participant's right arm from the home angle to a reference angle. The reference angles were 30° or 50° and chosen pseudo-randomly for each trial. Participants were instructed to relax and remember the reference angle. The elbow joint was then returned to the home angle by the robot, and then the elbow joint was flexed to the test angle. The test angles deviated from the reference angle by ± 2°, ± 4°, ± 6°, or ± 8° in each trial in a pseudo-random fashion. In this study, Two-Alternative Forced Choice (2AFC) was used, and the participants were asked to verbally indicate whether the test angle was smaller or larger than the reference angle. Note that this task also has a short-term memory component. However, the proprioceptive judgment task requires participants to memorize only a single angle and to discriminate subtle differences, from 2 to 8°, between the elbow joint angles presented sequentially. Therefore, this task is more demanding on proprioceptive discrimination than on short-term memory. By contrast, the short-term memory task is less sensitive to proprioceptive acuity due to the significant angular differences (> 10°) between the angles presented but more demanding on memory due to the multiple (three) angles to be memorized.

We calculated the proprioceptive acuity for each participant as follows. First, we calculated the probability of responding to the test angle as more flexed than the reference angle as a function of the angles of deviation (angle =  ± 8, ± 6, ± 4, ± 2°) from the reference angle. The probability was fitted with a cumulative Gaussian distribution function. We obtained the Just Noticeable Difference (JND) by calculating half the difference between the stimulus values (Xs: deviation angles in this experiment) corresponding to the probabilities of 75% and 25% in the fitted cumulative Gaussian distribution (JND = $\frac{{x}_{75}-{x}_{25}}{2}$)^19,20^. We considered the JND an index of proprioceptive acuity: A low JND indicates high acuity, whereas a high JND indicates low acuity. The average JND across participants was 4.24° (SD = 2.19°).

### Passively guided motor learning

The passively guided motor learning task was similar to the task used in our previous study^3^. This task consisted of four periods: a short instruction period, a pre-learning test, a long instruction (passive motor learning) period, and a post-learning test (Fig. 3a). In the short instruction period, the exoskeleton robot moved the participant’s forearm five times to show the target trajectory of the elbow joint movement. Following this period, each participant actively reproduced the target trajectory five times in the pre-learning test as their baseline reproduction performance. Next, in the long instruction (passive motor learning), the robot moved the participant’s forearm according to the target trajectory 30 times (passive physical guidance). Finally, in the post-learning test, participants reproduced the trajectory five times. We used the target trajectory shown in Fig. 3b for all participants to equalize the learning difficulty across participants.

**Figure 4:**
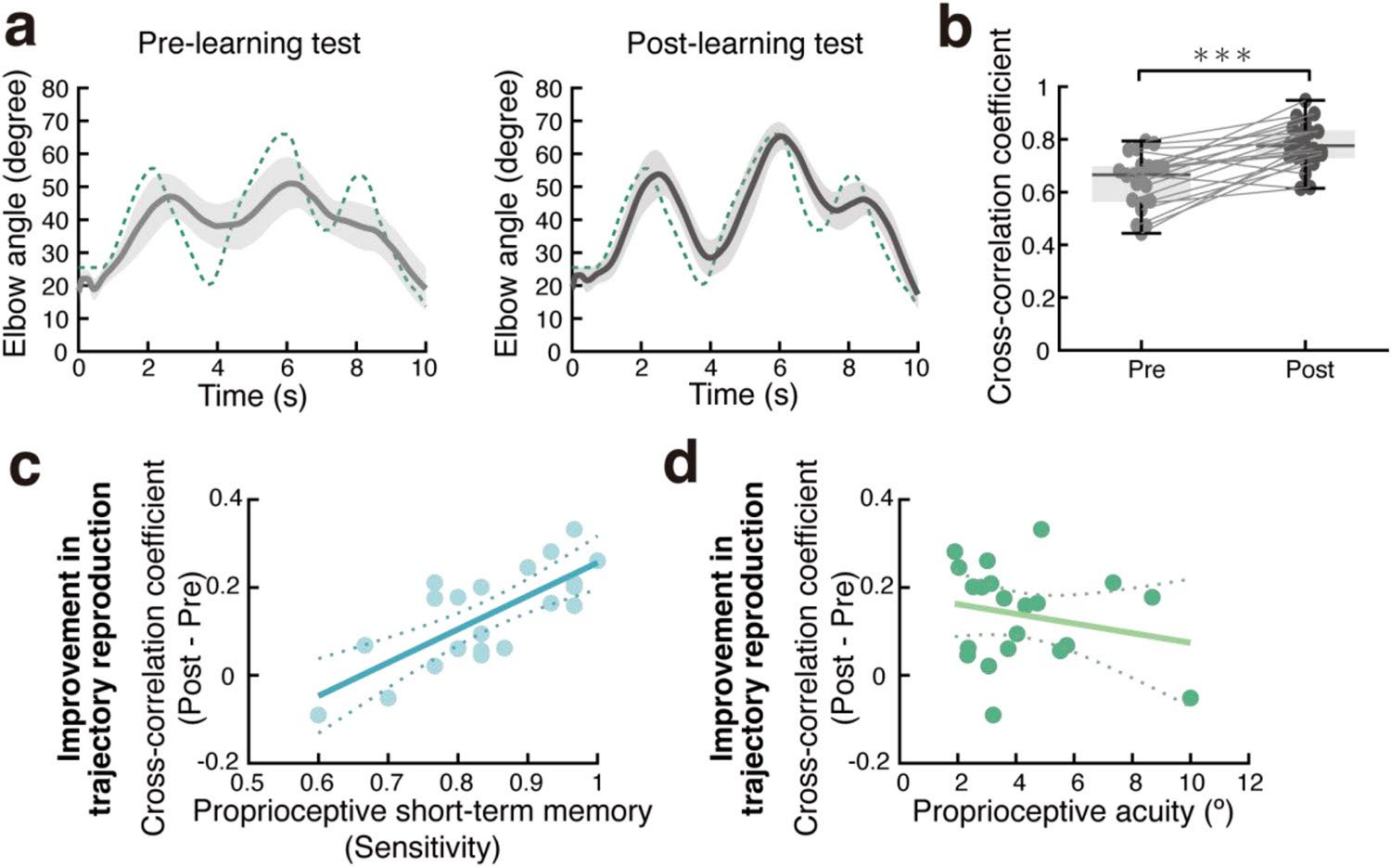
Improvement in trajectory learning and its relationship to proprioceptive task performance. (**a**) Reproduced trajectories of 10-s elbow movements in the pre-learning (left) and post-learning (right) tests. The mean (dark gray) and 95% CI (light gray) across participants are shown. Green dotted lines indicate the target trajectory (reference). (**b**) Mean cross-correlation coefficients between the target and reproduced trajectories in the pre-learning and post-learning tests. A circle indicates the performance of each participant. (**c**) Relationship between proprioceptive short-term memory and improvement in trajectory reproduction (Post–Pre). (**d**) Relationship between proprioceptive judgment and improvement in trajectory reproduction. Circles represent individual participants. Lines represent the regression line, and dotted lines represent the 95% CI.

Figure 4a shows the reproduction performance averaged across participants in the pre-learning and post-learning tests. The post-leaning test showed a clear improvement in reproducing the target trajectory compared to the pre-learning test. We calculated a cross-correlation coefficient to measure the similarity between the target trajectory and the reproduced trajectory in each pre- and post-learning trial. We defined a cross-correlation coefficient as the peak correlation calculated by the normalized cross-correlation function, a time-dependent Pearson correlation coefficient. The results show significantly higher correlation values for the post-learning test than for the pre-learning test [paired t-test: t(20) = 5.715, p < 0.001] (Fig. 4b). These results demonstrate the learning effect on trajectory reproduction due to passive guidance by the robot.

As an index of the learning effect of passive physical guidance, the degree of improvement in the trajectory reproducibility was calculated by subtracting the cross-correlation coefficient in the pre-learning test from that in the post-learning test for each participant. An index value greater than 0 indicates that trajectory reproducibility is better in the post-learning than in the pre-learning test. Our results reveal that trajectory reproducibility in fact improved in the post-learning test [mean = 0.14, SD = 0.11; *t*(20) = 5.715, *p* < 0.001].

### Correlation between short-term proprioceptive memory and passively guided learning

To investigate whether short-term proprioceptive memory is related to the learning efficacy of passively guided movements, we calculated the correlation between the proprioceptive short-term memory performance (sensitivity, precision, or d') and the degree of improvement in trajectory reproducibility (post-learning test–pre-learning test; see above). As a result, the improvement in trajectory reproducibility was significantly correlated with sensitivity (r = 0.75, p < 0.001; Fig. 4c) and d′ (r = 0.64, p = 0.002; Supplementary Fig. S1a) of proprioceptive short-term memory but not significantly correlated with precision (r = 0.23, p = 0.324; Supplementary Fig. S1b). These results suggest that participants with better sensitivity to memory angles showed better improvement in reproducing the passively guided trajectory.

### Correlation between proprioceptive acuity and passively guided learning

To investigate whether proprioceptive acuity is related to the learning effects of passive physical guidance, we calculated the correlation between proprioceptive acuity (JND) and the degree of improvement in trajectory reproduction. Figure 4d shows the relationship between the JND and trajectory learning. We did not find a significant correlation between the JND and improvement in trajectory reproducibility [r = − 0.218, p = 0.343].

### Chronological change in the correlation between short-term memory performance and passively guided learning

**Figure 5:**
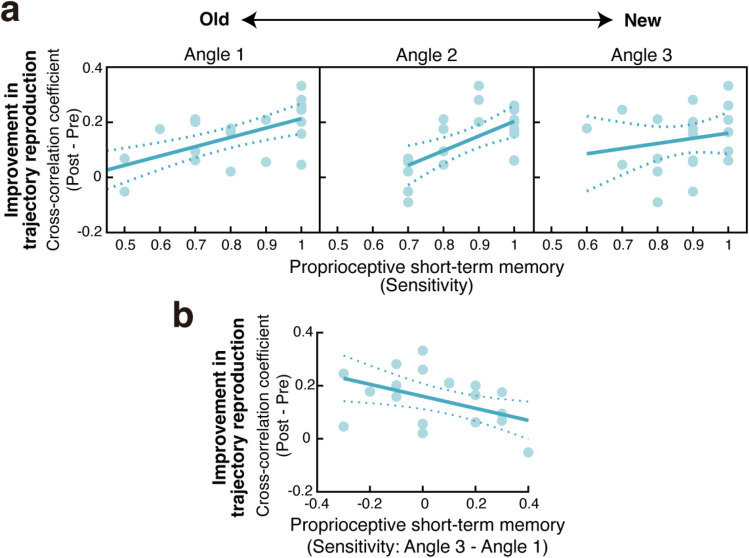
Chronological change in the relationship between short-term memory performance and improvement in trajectory reproduction. (**a**) Scatter plots show the relationship between short-term memory performance (sensitivity) and improvement in trajectory reproduction. Three panels show the relationships for the three memory angles chronologically. (**b**) A scatterplot showing the relationship between the memory preference for the temporal order of sensory experiences (sensitivity for the most recent item [angle 3] − sensitivity for the oldest item [angle 1]) and improvement in trajectory reproduction. Participants who remembered the old item more significantly improved trajectory reproduction. Circles represent individual participants. Lines represent the regression line, and dotted lines represent the 95% CI.

We examined whether the correlation between short-term memory performance and passively guided learning changed depending on the chronological order of the memory items (Angle 1: old, Angle 2: middle, Angle 3: new). The cross-correlation coefficient between the memory and the learning performance was r = 0.673 (p < 0.001) for Angle 1, r = 0.609 (p = 0.003) for Angle 2, and r = 0.192 (p = 0.403) for Angle 3. That is, the correlation coefficient decreased as a function of the temporal order of the memory items (Fig. 5a). This result suggests that participants who remembered older sensory experiences better than more recent ones were better able to learn from passive physical guidance. To quantify individual memory preference for temporal order, we subtracted the short-term memory performance (sensitivity) of Angle 1 from that of Angle 3 (Fig. 5b). Positive values for this index indicate that participants remember recent sensory experiences better than old ones, and negative values indicate that participants remember old sensory experiences better than recent ones. A significant negative correlation (r = − 0.508, p = 0.019) was observed between this index and improvement in the trajectory reproduction. In other words, participants who remembered their old sensory experiences better had a better learning effect.

### Influences of proprioceptive memory and acuity on passively guided learning

**Figure 6:**
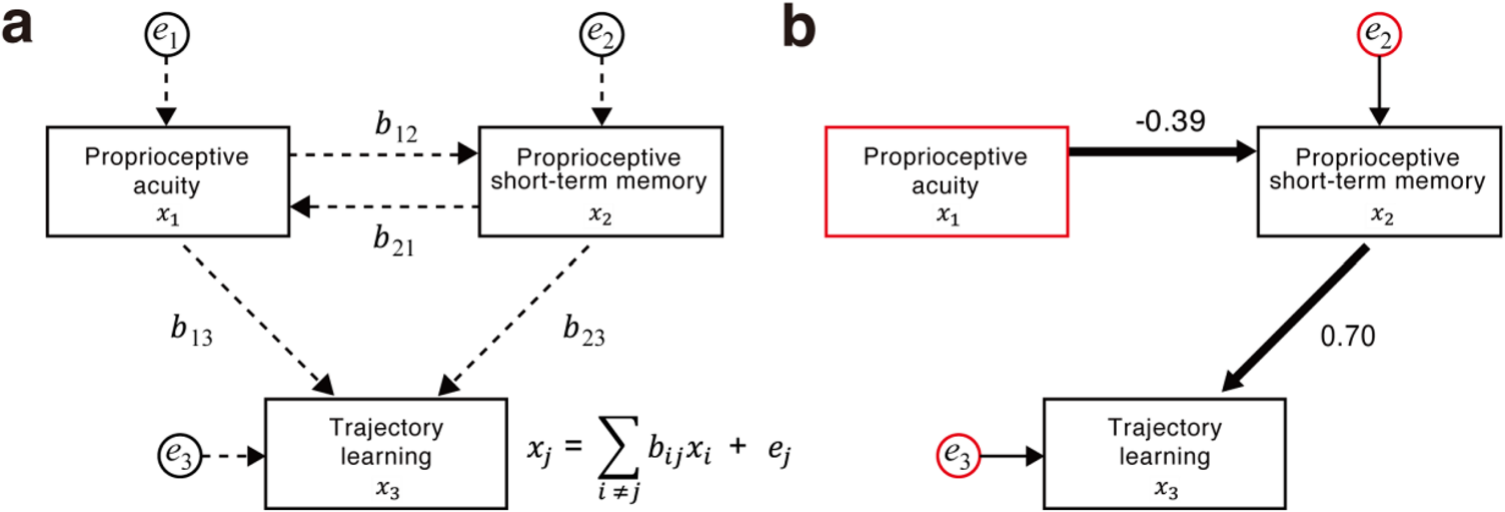
Effective acyclic graph estimated by DirectLiNGAM. (**a**) Causal structure of the baseline model. (**b**) Effective acyclic graph and causal effects estimated by DirectLiNGAM. Red frames indicate exogenous variables not determined by other variables within the estimated model.

We used a causal inference approach to examine the influence of short-term proprioceptive memory and proprioceptive acuity on improving trajectory reproduction. The causal structure of our baseline model hypothesized that both short-term proprioceptive memory and proprioceptive acuity influence the improvement in trajectory reproduction and that short-term memory and proprioceptive acuity also influence each other (Fig. 6a). A causal graph corresponding to this model was estimated using LiNGAM (linear non-Gaussian acyclic model)^16^, a semi-parametric approach algorithm. The LiNGAM coefficient matrix was calculated using DirectLiNGAM^21,22^, which estimates the causal order of the observed variables through repeated regression analysis and independent assessment.

As a result, the causal effect from short-term proprioceptive memory to improvement in trajectory reproduction was 0.70, and that from proprioceptive acuity to short-term memory was − 0.39 (note that smaller acuities are better). DirectLiNGAM uses a sparse regression method (Adaptive LASSO^23^) to estimate regression coefficients (*b*s in Fig. 6a). The sparse method estimated the coefficient as zero (no direct path) for the path from proprioceptive acuity to improvement in trajectory reproduction (Fig. 6b). The LiNGAM approach assumes independence and non-Gaussianity of exogenous variables (outlined in red in Fig. 6b) that are not determined by other variables within the model. First, we confirmed that the estimated model met both assumptions (see Methods for details). Then, we assessed the statistical reliability of the estimated causal paths by combining DirectLiNGAM with a bootstrap method^24,25^. The bootstrap probabilities of the estimated causal effects were 99.5% for the path from the short-term proprioceptive memory to the improvement in trajectory reproduction and 48.8% for the path from the proprioceptive acuity to short-term memory (Supplementary Table 1). Thus, the bootstrap method suggests that the effect of short-term memory is more reliable than that of proprioceptive acuity for improving trajectory reproduction.

Since the bootstrap probability of the effective acyclic graph estimated by LiNGAM (Fig. 6b) was relatively low (33.3%), we re-evaluated the graph structure using a structural equation model (SEM). As a result, the SEM fit indices were better than the standard goodness-of-fit values. This result indicates that the model closely represents the data (Supplementary Table S2). The strength was 0.75 for the path from short-term memory to improvement (z = 5.22, p < 0.001) and − 0.39 for the path from proprioceptive acuity to reproduction improvement (z = − 1.96, p = 0.050) (Supplementary Fig. S4). Therefore, the SEM analysis supported the LiNGAM result that the learning effect was higher for those with a better ability to remember the induced proprioception than those with better proprioceptive acuity.

## Discussion

This study tested the hypothesis that an individual's proprioceptive short-term memory capacity is associated with learning efficiency in passively guided training. As a result, participants with better short-term memory were more efficient at learning an elbow joint trajectory passively guided by an exoskeleton robot (Fig. 4c). In addition, participants with better memory for temporally remote items improved the reproduction of the passively guided trajectory (Fig. 5). We examined the individual’s proprioceptive acuity as a factor that may influence learning efficiency in passively guided motor learning. However, we did not find a significant correlation between acuity and learning efficiency (Fig. 4d). This result is consistent with our causal inference regarding the influence of memory and proprioceptive acuity on passively guided learning. We found a significant direct influence of memory, but not acuity on learning (Fig. 6b). However, acuity directly influences memory and indirectly influences learning via memory. These results highlight the crucial role of proprioceptive short-term memory in motor learning through passive physical guidance.

The improvement in trajectory reproduction was more strongly correlated with memory performance for elbow joint angles presented at an early stage than for those presented more recently (Fig. 5). There are two possible explanations for this finding. First, repetitions of similar stimuli to be remembered, such as the proprioceptive stimuli with subtle differences used in this study, are likely to be challenging to recall due to overwriting^26–28^. Participants who can overcome memory overwriting by new stimuli and thus remember older sensory stimuli are likelier to retain sensory information instructed by passive motion guidance. Second, for a continuous movement of a certain duration, such as the target trajectory in the current study, an error in the movement's early phase will affect the movement's subsequent phase compared to an error in the late phase. Therefore, participants who minimized the initial reproduction error with their accurate memory could also reduce this error over the duration of the movement. These factors may have contributed to the relative importance of short-term memory for early rather than late elements in movement reproduction.

We examined both sensitivity (hit/(hit + miss)) and precision (hit/(hit + false alarm)) as indices of short-term memory performance. As a result, improvement in the reproduction of the passively guided motion was associated with sensitivity rather than with precision. Sensitivity is the probability of correctly identifying the memorized angle when the test angle is one of the memorized angles. A sensitivity of 100% indicates that a participant never missed the test angle, which was one of the three memorized angles. In contrast, precision is the probability of correctly identifying the memorized angle when the participant responded that the test angle was one of the memorized angles. A precision of 100% indicates that the participant made no false alarms. Sensitivity, that is, the ability to avoid missing important information, is essential for passively guided training where a trainer or robot will likely teach the learner the correct movements.

We could not identify a significant relationship between proprioceptive acuity and improvement in trajectory reproduction (Fig. 4d). Furthermore, our causal inference using LiNGAM failed to identify a direct pathway from proprioceptive acuity to improvement in trajectory reproducibility (Fig. 6b). No conclusion can be drawn from these negative results. However, a possible reason for these results is that proprioceptive acuity only slightly affected our trajectory reproduction task. That is, we repeated the passive guidance by the robot many (30) times. Thus, even participants with poor proprioceptive acuity may compensate for their deficient acuity by accumulating sensory information during repetitive guidance^29^. Indeed, the influence of proprioceptive acuity on motor control and learning has been a point of controversy in previous studies. On the one hand, motor control and learning are impaired in patients with proprioceptive loss^30–33^, suggesting that proprioception is essential for the maintenance and formation of internal models of motor control^34^. On the other hand, previous studies in older adults have found little effect of age-related declines in sensory acuity on motor control or learning^35–37^. One of these studies suggests that healthy older participants may compensate for their proprioceptive decline by increasing their reliance on predictive models^36,37^. Therefore, it is easier to detect the effect of sensory acuity on motor performance with precise experimental control of the compensation for poor acuity.

It has been suggested that passive motor learning improves proprioceptive acuity^3,38,39^ while proprioceptive training improves motor learning^40,41^. Accordingly, proprioceptive acuity and motor learning interact^42^. However, our current study measured individual proprioceptive acuity only before motor learning. Therefore, it is unknown whether the improvement of proprioceptive acuity by a passive motor learning task affects efficacy in the same learning task. It might be necessary to repeat the measurement of acuity and motor learning several times to investigate their interaction in the current experimental paradigm. Moreover, the interaction between proprioceptive short-term memory and motor learning is also unknown. Consequently, future studies must reveal reciprocal improvement mechanisms between acuity and learning and between short-term memory and learning.

We calculated the cross-correlation between the target trajectory and the participant's reproduced trajectory to assess trajectory reproduction performance. We used the highest correlation value across the temporal shift of one trajectory relative to the other. Thus, we ignored the temporal difference between the two trajectories. In our additional analysis, we calculated the root mean square error (RMSE), including the temporal difference. Results using RMSE were consistent with those using cross-correlation concerning our main findings (Supplementary Fig. S2): (1) a significant improvement in trajectory reproduction [a paired t-test: t(20) = 3.713, p = 0.001], (2) a significant correlation between improvement in trajectory reproduction and proprioceptive short-term memory sensitivity [r = 0.44, p = 0.048], and (3) no significant correlation between reproduction improvement and proprioceptive acuity [r = 0.10, p = 0.657]. In contrast, using the RMSE, we could not statistically confirm our finding that participants with better memory for temporally remote items showed better reproduction improvement (r = − 0.22, p = 0.344, Supplementary Fig. S3). The inconsistent results between the RMSE and cross-correlation probably reflect the finding that the participants attended to the spatiotemporal pattern of the target trajectory but not to the onset time of the trajectory reproduction. Our primary analysis disregarded the onset time by instead taking the highest value of the cross-correlation function.

Finally, we must point out several limitations of our current study. First, as mentioned above, our finding of no significant correlation between the improvement in trajectory reproducibility and proprioceptive acuity requires further investigation. Second, our causal inference model based on DirectLiNGAM showed a relatively low bootstrapping probability (33.3%). Thus, by using SEM, we confirmed the statistical significance of the paths found by the DirectLiNGAM analysis: the path from proprioceptive short-term memory to improvement in trajectory reproduction (z = 5.22, p < 0.001) and the path from proprioceptive acuity to short-term memory (z = − 1.96, p = 0.050). However, these results are based on correlations between behavioral measures across individuals. Therefore, we must verify our causal inference through future within-participant experimental manipulations. For example, we can test whether proprioceptive short-term memory training would increase trajectory reproducibility compared to a no-training control group to verify the pathway from memory to improved trajectory reproduction.

This study investigated the relationship between the learning effect of passive training, proprioceptive short-term memory, and proprioceptive acuity. The results show that the learning effect of passive physical guidance is higher for those who can retain past sensory information. Our results demonstrate the importance of proprioceptive short-term memory in passively guided physical training. Robotic rehabilitation using passively guided training has recently gained importance for automated physical therapy that provides consistent training. Accordingly, the current study suggests that the client’s proprioceptive short-term memory capacity must be considered in developing practical passive training. In a broader context, it is often the case that the timing of the learner's performance is separated from the presentation of the model to be imitated by the learner. For example, in pronunciation learning, the ideal pronunciation is presented by a teacher and then imitated by the learner. The learner's acoustic sensitivity and short-term memory are also expected to be essential for efficient learning. The combination of the experimental paradigm and causal inference proposed in this study could help to investigate the relationships among sensitivity, short-term memory, and learning efficiency in a variety of learning domains.

## Methods

### Study participants

We recruited 21 healthy adults. The mean age was 25.6 years (19–38 years). All participants were right-handed to control for the effects of handedness on elbow movements and perception. None of the participants had a history of visual impairment, neurological disease, or musculoskeletal dysfunction. Informed consent was obtained from all participants before the start of the experiment. All experiments were approved by the Ethics Committee of the Advanced Telecommunications Research Institute International, Kyoto, Japan, and were conducted in accordance with the Declaration of Helsinki. A person in the photograph (Fig. 2b) is the one of authors (not included in our participants) representing how participants’ arms are attached to the exoskeleton robot. He has agreed to publish the images in an online open-access publication.

### Exoskeleton robot

We used the upper limb exoskeleton robot^17,18^, which is characterized by its back-drivable joints with low inertia links. The exoskeleton’s joints were driven by electric motors with low-gear rations. This design allows the user to move the forearm freely when the motors are not engaged. The link lengths of the exoskeleton robot are 0.265 m from the shoulder to the elbow joint and 0.26 m from the elbow to the wrist, and it has four degrees of freedom: shoulder flexion/extension, shoulder abduction/adduction, elbow flexion/extension, and wrist flexion/extension joints. In this study, only the flexion/extension of the elbow joint was moved by an electric actuator in all experiments. The shoulder joint was fixed in the horizontal plane by a pin, and the shoulder joint was set at an angle of 70° of horizontal flexion by pneumatic artificial muscles (Fig. 1a). The participant's arm was attached to the exoskeleton arm by Velcro straps between the shoulder and elbow, and between the elbow and wrist. The participants were asked to lightly grip the hand strap to prevent the finger position from changing.

### Proprioceptive short-term memory task

A short-term memory task was used to investigate whether short-term proprioceptive memory is related to the learning efficiency in passively guided movements. The total number of trials was 60. In each trial, three memory angles were pseudo-randomly selected from the 38 angles between 8° and 82° (at intervals of 2°). In half of the total trials (30 trials), the test angle was one of the three memory angles where the correct response was “yes.” In the remaining 30 trials, the test angle was a new angle that differed by more than 10° from one of the three memory angles. Thus, the correct response was “no” in these trials. The new angle was also chosen pseudo-randomly from angles between 8º and 82° (at intervals of 2°). The elbow angles presented in each trial were selected so that each angle differed by at least 10° from the other angles to avoid the influence of the perceptual discrimination ability of proprioception.

### Data analysis

#### Trajectory performance analysis

The elbow joint angle was measured at a rate of 250 Hz using an encoder attached to an exoskeleton robot. We calculated the cross-correlation coefficient (Fig. 4b) and RMSE (Supplementary Fig. S2) between the target and reproduced trajectories of the elbow joint. We used a paired t-test to investigate whether the reproducibility of the motor trajectories changed before and after the long instruction (passive motor learning).

#### DirectLiNGAM

DirectLiNGAM^21,22^ was used to explore the causal relationships between three variables (trajectory learning, proprioceptive short-term memory, and proprioceptive acuity). We used the LiNGAM package (https://github.com/cdt15/lingam)^43^. The causal structure in our baseline model is shown in Fig. 6a. The estimated causal graph equation is

$${x}_{i}={\sum }_{j \ne i}{b}_{ij}{x}_{j} + {e}_{i},$$

where x is the observed variable, b is the path coefficient, and e is the error variable. Standardization (z-score normalization) was performed for each variable to avoid the influence of each variable’s units. Here, we examined violations of the assumptions of the LiNGAM approach: independence and non-Gaussianity of the exogenous variables (outlined in red in Fig. 6b). First, we examined the independence of the estimated exogenous variables using a nonparametric independence measure, the Hilbert–Schmidt independence criterion test (HSIC). As a result, the independence assumption was not rejected for any of the following pairs of variables: (1) trajectory learning error variable and proprioceptive short-term memory error variable (p = 0.783), (2) trajectory learning error variable and proprioceptive acuity (p = 0.480), and (3) proprioceptive short-term memory error variable and proprioceptive acuity (p = 0.720). Next, we used the Shapiro–Wilk test to test the non-Gaussianity of the exogenous variables. We confirmed non-Gaussianity in proprioceptive acuity (W = 0.86, p = 0.006) and the error variable for trajectory learning (W = 0.90, p = 0.034). However, we did not find significant support for the error variable for proprioceptive short-term memory (W = 0.94, p = 0.258). Note that the causal graph is identifiable even when one of the exogenous variables lacks non-Gaussianity. These results suggest that our estimation is consistent with the assumptions of the LiNGAM approach. To calculate the probability of the estimated causal graph and paths, we performed bootstrapping with 3,000 resamples.

### Structural equation model (SEM) path analysis

To test the validity of the model estimated by DirectLiNGAM, we applied an SEM analysis to our data, with the causal structure estimated by DirectLiNGAM. We assessed the goodness of fit of the SEM using four indices (Supplementary Table S2). According to the references, each index value was higher than the standard value^44–47^. This result suggests that the SEM analysis supports the causal structure estimated by DirectLiNGAM.

### Supplementary Information

Supplementary Information.

## Data Availability

The data reported in this paper are available from the corresponding author upon reasonable request.
