# Supplementary material for: Proprioceptive short-term memory in passive motor learning
Source: Sci Rep. 2023 Nov 27;13:20826. doi: 10.1038/s41598-023-48101-9 (PMC10682388; doi:10.1038/s41598-023-48101-9)
Supplement: Supplementary file 1 — Supplementary Information. [file 41598_2023_48101_MOESM1_ESM.docx]

**Supplementary Information**

**SI Figures**


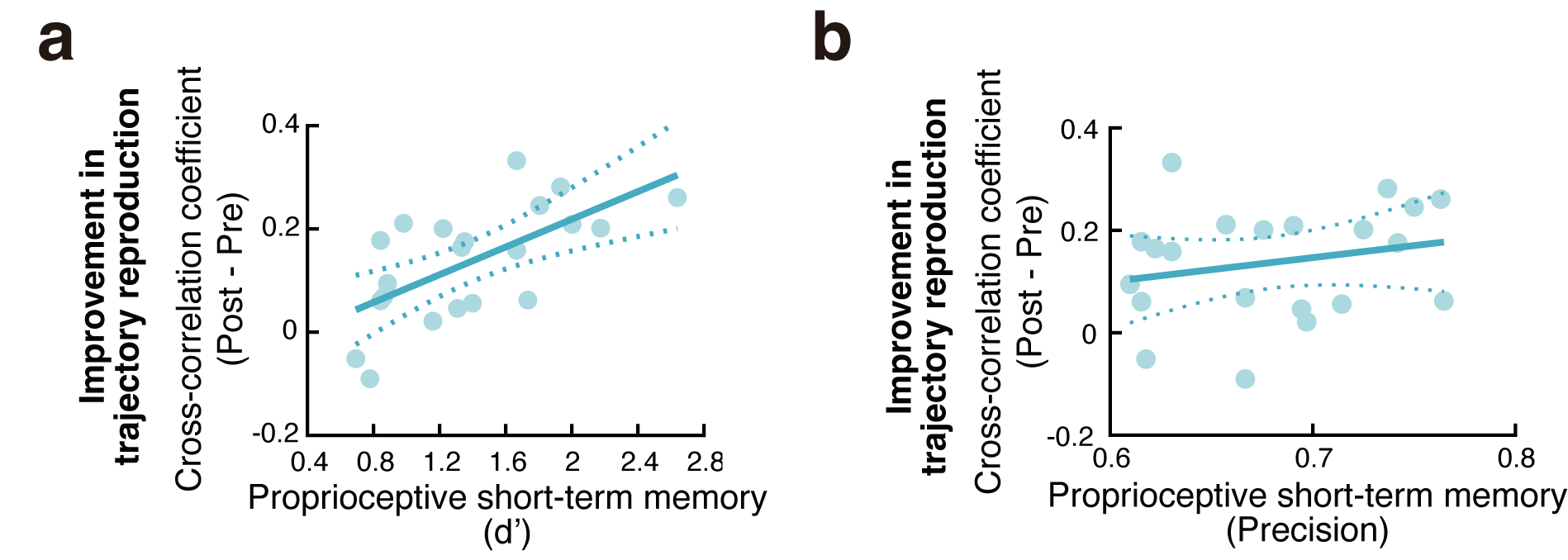


**Supplementary Figure S1. Relationship between proprioceptive short-term memory performance (d’ (a) or precision (b)) and improvement in reproduction performance.** Scatter plots show the relationship between short-term memory performance and improvement in trajectory reproduction. Reproduction performance was calculated using the cross-correlation coefficient between the target and reproduced trajectories. Proprioceptive short-term memory performance was calculated using d’ (a) or precision (b). Improvement in trajectory reproduction was significantly correlated with d' (r = 0.64, p = 0.002) but not significantly associated with precision (r = 0.23, p = 0.324). Dots indicate participants. Solid and dotted lines indicate the regression line and 95%CI, respectively.


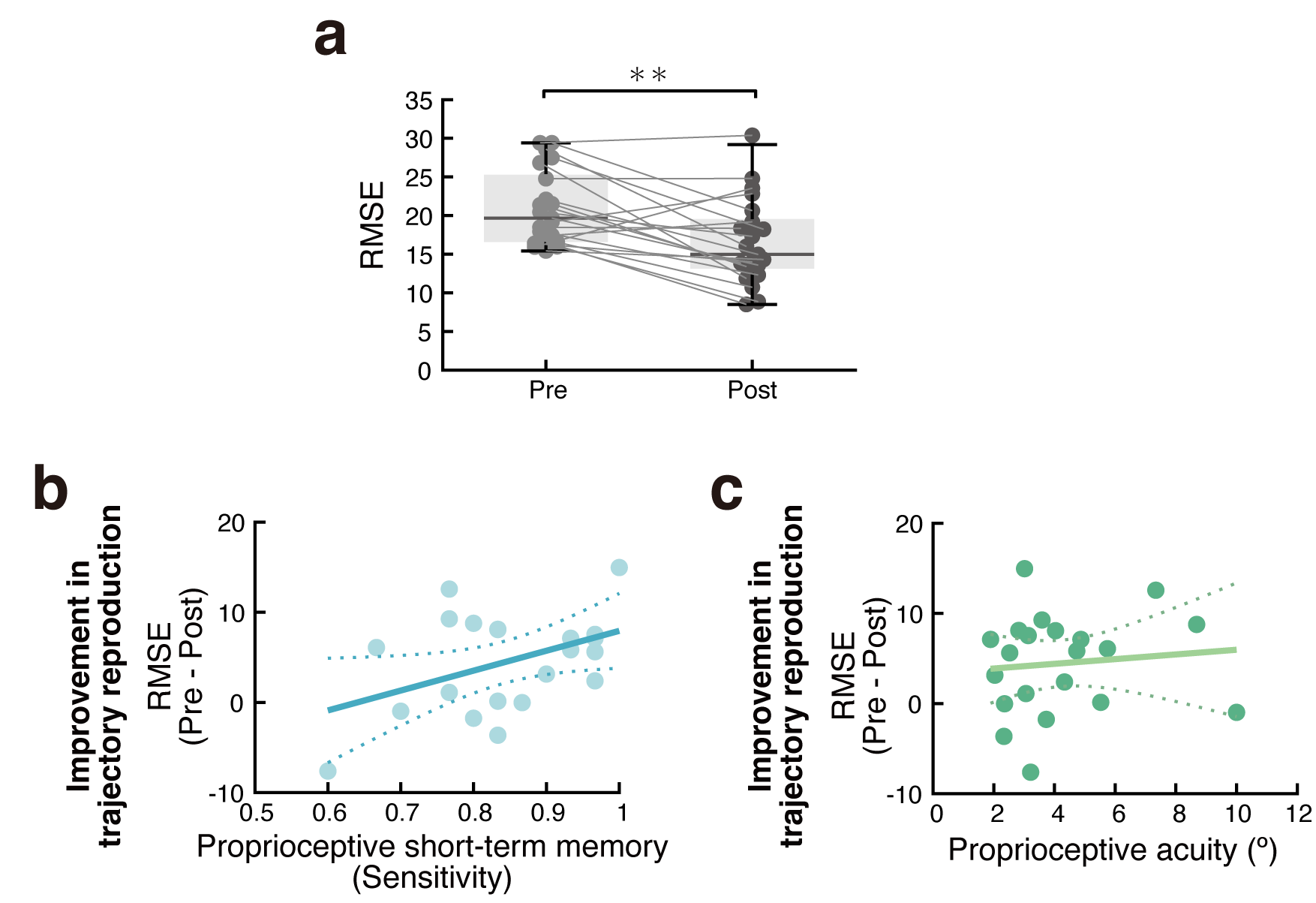


**Supplementary Figure S2. Relationship between trajectory learning (RMSE) and proprioceptive task performance.** (a) Trajectory reproduction performances in the pre-learning and post-learning tests calculated by RMSE. Dots represent individual participants. We found a significant decrease in RMSE from the pre-learning to the post-learning test [paired t-test: t(20) = 3.713, p = 0.001]. (b) Scatterplot showing the relationship between proprioceptive short-term memory and improvement in reproduction performance (pre-learning test – post-learning test). We found a significant correlation between the measures [r = 0.44, p = 0.048]. (c) Scatter plot showing the relationship between proprioceptive acuity and improvement in reproduction performance. Dots represent individual participants. We did not find a significant correlation between the measures [r = 0.10, p = 0.657]. Solid and dotted lines represent the regression line and the 95%CI, respectively.


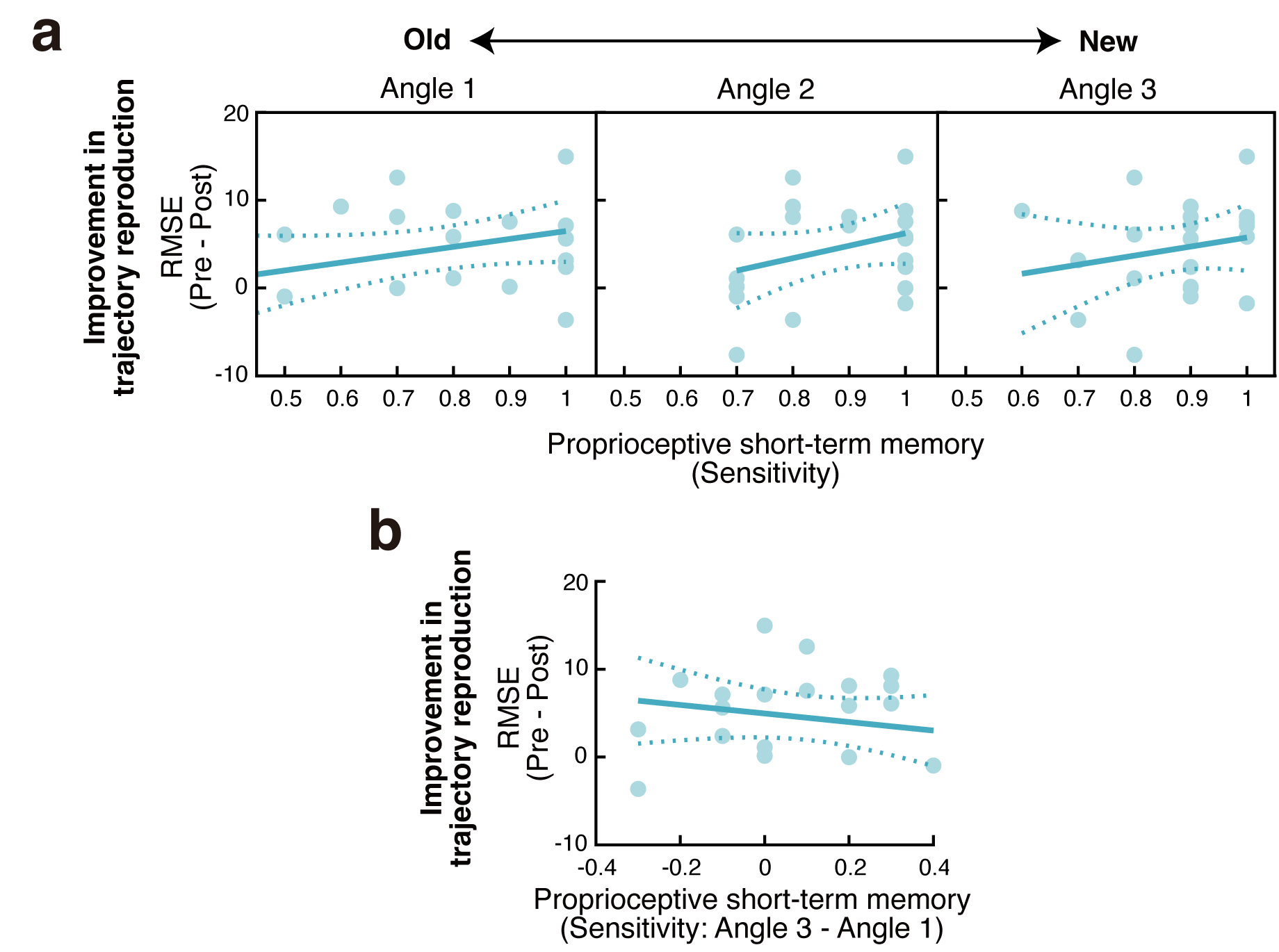


**Supplementary Figure S3. Chronological change in the relationship between proprioceptive short-term memory performance and trajectory reproduction (RMSE) improvement.** (a) Scatterplots show the relationship between short-term memory performance (sensitivity) and improvement in trajectory reproduction. Three panels show the relationships for the three memory angles chronologically. (b) Scatterplot showing the memory preference for the temporal order of sensory experiences (sensitivity for the most recent item [angle 3] – sensitivity for the oldest item [angle 1], i.e., plus values indicate preferences for recent items) and improvement in trajectory reproduction. When we assessed trajectory reproduction performance using RMSE, we did not find a significant relationship between the memory preference for the temporal order and improvement in trajectory reproduction. Dots represent individual participants, solid lines indicate regression lines, and dotted lines indicate 95% CI.

**Supplementary Table S1.** **Bootstrap probability of causal effects**

| Causal direction | | | Effect | Probability |
| --- | --- | --- | --- | --- |
| Proprioceptive short-term memory | → | Trajectory learning | 0.727 | 0.994 |
| Proprioceptive acuity | → | Proprioceptive short-term memory | -0.447 | 0.488 |
| Proprioceptive acuity | → | Trajectory learning | -0.438 | 0.253 |
| Proprioceptive short-term memory | → | Proprioceptive acuity | -0.600 | 0.076 |

**Supplementary Table S2. The goodness of fit for SEM**

|  | GFI | AGFI | RMSEA | CFI |
| --- | --- | --- | --- | --- |
| Model estimated by LiNGAM | 0.984 | 0.951 | 0.000 | 1.000 |
| Standard of good fit | > 0.95 | > 0.95 | < 0.05 | > 0.95 |

Note: GFI: Goodness-of-fit index; AGFI: Adjusted GFI; RMSEA: Root mean square error of approximation; CFI: Comparative fit index


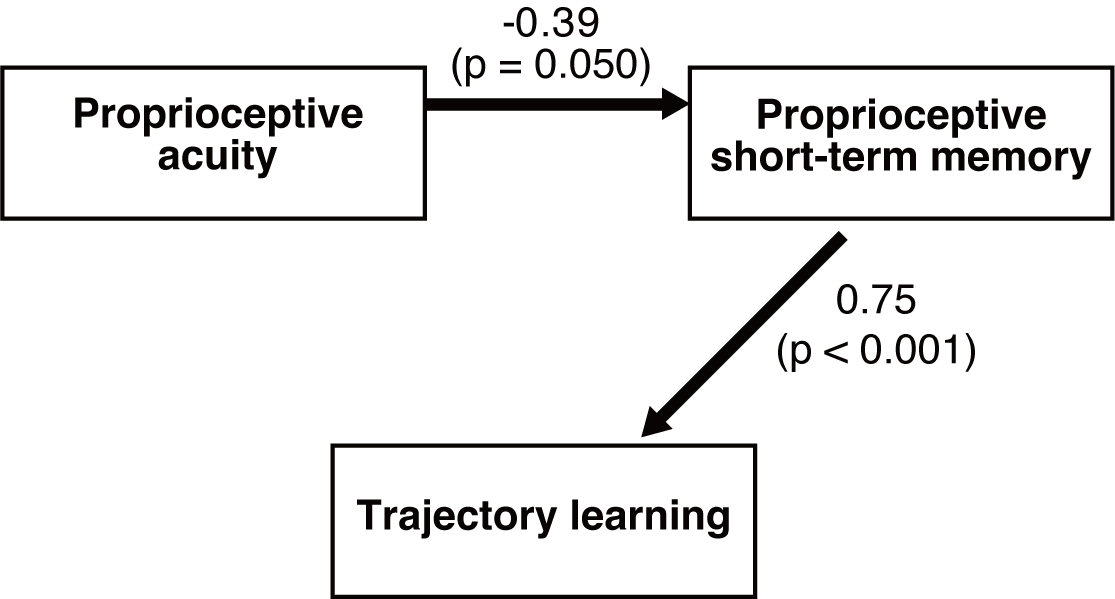


**Supplementary Figure S4. Path diagram by SEM.** Re-evaluation of causal graph estimated by LiNGAM using SEM. The SEM results supported the LiNGAM results (Figure 6 in main text).
